# Changes in Salivary Proteome in Response to Bread Odour

**DOI:** 10.3390/nu12041002

**Published:** 2020-04-05

**Authors:** Laura Carreira, Paula Midori Castelo, Carla Simões, Fernando Capela e Silva, Cláudia Viegas, Elsa Lamy

**Affiliations:** 1 MED—Mediterranean Institute for Agriculture, Environment and Development, University of Évora, 7002-554 Évora, Portugal; m39592@alunos.uevora (L.C.); carlasimoes3@hotmail.com (C.S.); fcs@uevora.pt (F.C.e.S.); 2 Department of Pharmaceutical Sciences, Institute of Environmental, Chemical and Pharmaceutical Sciences, Universidade Federal de São Paulo (UNIFESP), São Paulo-SP 04021-001, Brazil; pcastelo@yahoo.com; 3 Department of Biology, School of Science and Technology, University of Évora, 7000-671 Évora, Portugal; 4 Department of Food Science, Estoril Higher Institute of Hospitality and Tourism (ESHTE), 2769-510 Estoril, Portugal; xviegas@icloud.com; 5 IIFA—Institute for Advanced Studies and Research, University of Évora, 7002-554 Évora, Portugal

**Keywords:** pre-ingestive signals, saliva, proteomics, food acceptance

## Abstract

It is widely recognized that smelling food results in a mouth-watering feeling and influences appetite. However, besides changes in volume, little is known about the effects that food odours have on the composition of saliva. The aim of the present study was to access the effects that smelling bread has on saliva proteome and to compare such effects with those of chewing and ingesting it. Besides a significant increase in saliva flow rate, together with a decrease in total protein concentration, bread odour induced changes in the proportion of different salivary proteins. The expression levels of two spots of cystatins and two spots of amylase increased due to olfactory stimulation, similar to what happened with bread mastication, suggesting that odour can allow anticipation of the type of food eaten and consequently the physiological oral changes necessary to that ingestion. An interesting finding was that bread odour increased the expression levels of several protein spots of immunoglobulin chains, which were decreased by both bread or rice mastication. This may be of clinical relevance since food olfactory stimulation of salivary immunoglobulins can be used to potentiate the oral immune function of saliva. Moreover, the effects of bread odour in the levels of salivary proteins, previously observed to be involved in oral food processing led to the hypothesis of an influence of this odour in the sensory perception of foods further ingested. Further studies are needed to elucidate this point, as well as whether the changes observed for bread odour are specific, or if different food odours lead to similar salivary proteome responses.

## 1. Introduction

Food sensory cues play a role in food acceptance and food choices in different ways. Associations between nutrient and energy content, as well as pleasure/comfort provided by foods are paired with their sensory characteristics. This learning will result in higher desire/consumption or in avoidance when such sensory signals are presented [1,2]. Different studies show the influence that the exposure to sensory cues has in wanting for food [3,4]. 

Among the pre-ingestive sensory cues, food odours are important modulators of appetite, influencing ingestive behaviour. Through smell, individuals can predict the characteristics of foods. For example, the exposure to banana odour increases a specific appetite for banana and for other sweet products [5]. Moreover, it was observed that besides sweet odours increasing appetite for sweet products, it may decrease appetite for savoury products, and vice-versa [6,7].

The anticipatory physiological responses to food odours (considered in the cephalic phase of response to ingestion) intend to prepare the body for ingestion and digestion of foods [8]. There is empirical and scientific evidence that the odour of a palatable food induces saliva secretion. Exposure to odours of chocolate or beef were observed to increase salivation compared to a situation of absence of odours [9] and a recent study demonstrated that this rise in salivary flow rate after food-odour stimulation does not occur in response to non-food odour [10]. 

Despite what was stated above, the influence of food odours exposure in the chemical composition of saliva is less known. Recently, Morquecho-Campos and colleagues [10] reported no significant effects of food odours in salivary protein composition, more specifically in salivary MUC5B, alpha amylase and lingual lipase enzymatic activities. However, as the authors discuss, no other changes in salivary proteome were assessed and changes in other salivary proteins are not discarded to occur in response to food odours.

Saliva is recognized by having different functions, with a major role in oral cavity defence and oral and systemic health. In recent years, the influence of saliva in oral food perception and the importance that this influence can have on food acceptance and preferences gained major interest. Salivary proteins such as proline-rich proteins (PRPs) [11,12] and cystatins [13,14] levels have been linked to different levels of perception and acceptance of astringent foods and beverages. Also, salivary carbonic anhydrase VI, cystatins and PRPs have been linked to the intensity of bitterness perception [15,16,17] and amylase, carbonic anhydrase VI and cystatins seem to be linked to sweet taste sensitivity [18]. Knowing how saliva varies in response to stimulants can be useful to understanding how they may modify subsequent food perception, information that can be used in nutrition programmes or in the catering and hotel industry.

Recently, in experiments relating the inter-individual variability of salivary response to bread mastication with differences in the way individuals perceived bread sensory aspects, it was observed that different salivary proteins can be related to bread sensory ratings (submitted). For example, salivary amylase amounts were found to relate negatively to bread sweetness and saltiness ratings. Moreover, amylase and cystatins seem to influence roughness perception, with salivary amylase contributing to higher and cystatins to lower roughness ratings (not published). 

The aim of the present study was to investigate the effects of bread odour stimulation in salivary proteome and, in the case of salivary changes, whether these are similar to those induced by general food mastication or even by the mastication of the specific type of food. Bread was the food chosen for testing, because this is well accepted by individuals and has a characteristic odour. Since bread is starch-rich and since salivary amylase, that is one of the most abundant salivary proteins, is involved in starch digestion, we hypothesized that some of the changes in saliva composition could be due to this nutrient. As such, we controlled the effect of starch by testing the mastication of rice, which is a food with very distinct sensory characteristics (in aroma).

## 2. Material and Methods

### 2.1. Participants

Twenty-five healthy women, 19–30 years old, participated in this study. The inclusion criteria were the absence of signs or symptoms of oral and systemic diseases and the absence of taking medication. Only 4 of the participants referred occasional smoking. No usual smokers were included. Prior to experiments, each participant was instructed not to eat nor drink anything, except water, for at least one hour and a half before the beginning of each session. Assays were all performed before 4:00 and 5:00 pm, to avoid circadian effects. 

Before the beginning of the study, all subjects read and signed an informed consent form. All procedures were performed according to the Declaration of Helsinki for Medical Research Involving Human Subjects and the study received approval from the Ethical Committee of the University of Évora.

### 2.2. Stimulation and Saliva Collection

Three types of stimuli were tested: (1) smelling bread, to assess the effect of food smell isolated from the other sensations; (2) chewing bread, to assess the effect of having the complex sensory stimulation of a normal ingestive process; (3) chewing boiled rice, to assess the effect of a product with sensory characteristics different from those of bread, but maintaining the same level of starch. This control was chosen, since starch is digested by salivary amylase and a direct effect of starch in the amounts of this salivary protein cannot be discarded.

**Figure 1:**
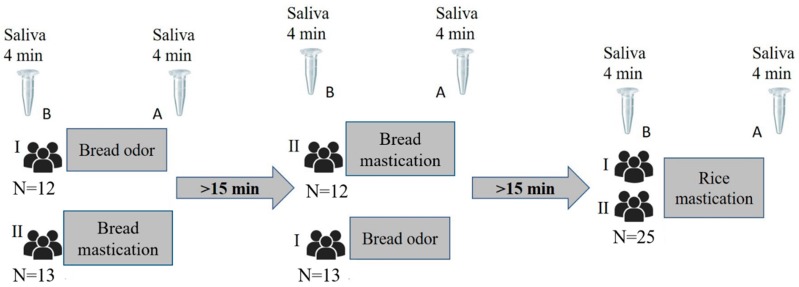
Schematic representation of practical experiment (I and II represent the two groups of participants, which collected saliva before (B) and after (A) each stimulus).

Stimuli order was randomly presented to the different individuals, for bread mastication and odour. Rice mastication was always the last stimuli tested. All of them were tested with the three stimuli and the interval between stimuli presentation was higher than 15 min (between 15 and 20 min) to avoid carry-over effect of previous stimulation. This time was chosen based on the results of an experiment previously performed in the laboratory, where it was observed that this period is long enough to delete possible saliva changes induced by chewing and deglutition of small amounts of food (*not published*). Saliva samples were always collected immediately before and during stimulation (in the case of smell) or immediately after chewing (in the cases of bread and rice). A schematic representation of the experiment is presented in Figure 1.

For bread smell stimulation, and in order to avoid any influence of other types of stimuli, despite individuals being informed that they would smell bread they were blindfolded and, only after that, were bread samples contained in a bowl presented. Individuals were kept separated to avoid any influence from one to another. For bread chewing stimulation, 10 g of bread (the same sample that was used to smell stimulation) was given to each participant. For rice chewing stimulation an amount of 19.8 g of boiled rice was given to each participant. This amount was estimated using the data from the Portuguese Food Composition Table (information available at http://portfir.insa.pt/) as reference to guarantee a starch administration equivalent in bread and rice samples.

Before each stimulus presentation, individuals drank water to eliminate any residual saliva and waited 30 s, after which they did not swallow during 4 min, spiting all of the saliva produced in the mouth to a clean polyethylene tube maintained in ice. This period was chosen both because it allows the collection of a saliva volume enough to laboratorial analysis and, at the same time, should allow effects of odour stimulation to be observed [10]. After this, the mouth was rinsed with water, and the participant was presented with the stimulus. In the case of bread smelling, the 4 min of saliva collection occurred with individuals keeping the bread near the nose, to assure that saliva produced during that time could have the effect of bread odour. In the case of bread or rice chewing, individuals were asked to slowly chew each of them and immediately after, drink a very small amount of water (only to remove major food residuals) and start collecting saliva to a new tube, using the same procedure described for the saliva collection before stimulation.

After experimental procedures, saliva samples were transported to the laboratory and stored frozen at −28 °C. In the days after collection, saliva samples were thawed on ice and centrifuged at 13,000× *g* 4 °C for 20 min, to precipitate insoluble material and recover homogeneous liquid samples.

### 2.3. Salivary Flow Rate and Total Protein Quantification

Saliva flow rate was assessed by assuming that saliva density is 1.0. Tubes containing saliva were weighed and the empty tube weight was subtracted. The final value was divided by 4 (minutes of collection). Total protein concentration was determined by the Bradford method, using bovine serum albumin (BSA) as standard, and plates were read at 600 nm in a microplate reader (Glomax, Promega, Madison, WI, USA).

### 2.4. Salivary Amylase Enzymatic Activity

A Salimetrics^®^ kit was used to determine the enzymatic activity of salivary amylase according to the manufacturer’s recommendations. Briefly, saliva samples were diluted 200× and applied on the microplate in duplicate, followed by application of a substrate (2-chloro-p-nitrophenol) preheated to 37 °C. The mixture was incubated at 37 °C for 1 min, absorbance values were read at 405 nm in a plate reader spectrophotometer, followed by incubation for an additional 2 min at 37 °C and a new reading at 405 nm. The enzymatic activity of amylase (U/ml) was calculated by the following formula: (ΔAbs./min × TV × DF)/(MMA × SV × LP), where ΔAbs./Min is absorbance variation per minute, TV is total test volume (0.287 mL), DF is dilution factor, MMA is millimolar absorbance of substrate 2-chloro-p-nitrophenol (12.9), SV is sample volume (0.007 mL), and LP is light path (0.97, specific for plate received with kit).

### 2.5. Sodium Dodecyl Sulphate-Polyacrylamide Gel Electrophoresis (SDS-PAGE) Salivary Protein Separation and Protein Band Identification

Each saliva sample was run in duplicate. For each sample, a volume corresponding to 6.5 µg total protein was mixed with sample buffer and run on each lane of a 14% polyacrylamide mini-gel (Protean xi, Bio-Rad, CA, USA) using a Laemmli buffer system, as described elsewhere [19]. An electrophoretic run was performed at a constant voltage of 140 V until front dye reached the end of the gel. Gels were fixed for 1 h in 40% methanol/10% acetic acid, followed by staining for 2 h with Coomassie Brilliant Blue (CBB) G-250. Gel images were acquired using a scanning Molecular Dynamics densitometer with internal calibration and LabScan software (GE Healthcare), and images were analysed using GelAnalyzer software (GelAnalyzer 2010a by Istvan Lazar, www.gelanalyzer.com) for the volume percentage of each protein band. Molecular masses were determined in accordance with molecular mass standards (Bio-Rad Precision Plus Protein Dual Colour 161-0394) run with protein samples.

The protein bands used for comparison among stimuli treatments were excised from well-resolved gels and tryptic digested with porcine trypsin (Sequencing Grade Modified Trypsin, Promega) following the protocol previously described elsewhere [19]. Protein identification was performed in a matrix-assisted laser desorption/ionization time-of-flight tandem (MALDI TOF-TOF) mass spectrometer (AB Sciex 4800 Plus) using 4000 Series Explorer v. 3.5.3.3 analysis software (Applied Biosystems, CA, USA). Samples were desalted and concentrated using reversed-phase Poros R2 (Applied Biosystems) and eluted directly to the MALDI target with matrix solution (α-cyano-4-hydroxycinnamic acid, CHCA; Fluka), which was prepared at a concentration of 10 µg/µL in 50% acetonitrile with 0.1% trifluoracetic acid (TFA). External calibration was executed using CalMix5 (Protea). The 30 highly intense precursor ions of MS spectra were selected for analysis by MS/MS.

The monoisotopic masses of the peptides were used to search for protein identification through the use of Protein Pilot v. 4.5 software (AB Sciex) with the Mascot search engine [MOWSE (MOlecular Weight Search) algorithm]. The Swiss-Prot database, restricted to *Homo sapiens*, was used for all searches. A minimum mass accuracy of 50 ppm and a mass tolerance of 0.3 Da, 2 missed cleavages in peptide mass, carbamidomethylation of Cys and oxidation of Met, as fixed and variable amino acid modifications, respectively, were considered. The criteria used to accept the identification were an homology score higher than 56 and at least one fragmented peptide with an individual significant score (*p* < 0.05) in Mascot.

### 2.6. Two-Dimensional Electrophoresis

In order to assess changes induced by bread odour and compare them with the effect of bread or rice chewing, 7 individuals were randomly selected to compare the salivary two-electrophoretic profiles of their samples. This number of individuals was chosen considering that this approach limits the number of samples to be run and analysed. Moreover, to minimize technical errors, each sample was run in duplicate. At the end, a total of 84 two-dimensional electrophoresis (2-DE) gels (7 individuals × 6 collection moments × 2 technical repetitions) was analyzed.

For 2-DE, each saliva sample (volume corresponding to 150µg of total protein) was concentrated and de-salted by centrifugation at 13,000× *g*, at 4 °C, in membranes with a cut-off of 3kDa. The centrifugation time was the one necessary to recover a volume of sample, in the upper reservoir, lower than 25 µL. The volume of the concentrated saliva sample was mixed with rehydration buffer [7 M urea, 2 M thiourea, 4% (*w*/*v*) CHAPS (3-[(3-Cholamidopropyl)dimethylammonio]-1-propanesulfonate hydrate), 2% (*v*/*v*), 60 mM DTT (Dithiothreitol) and traces of bromophenol blue] +2.5 µL IPG buffer to achieve a final volume of 125 µL. The mixture was incubated during 1h at room temperature, being subsequently centrifuged for 10 min at 10,000 rpm. IPG strips (7 cm, pH 3–10 NL; GE, Healthcare) were passively re-hydrated overnight with this solution. Focusing was performed in a Multiphor II (GE, Healthcare) at 18 °C, with the programme (gradient): (1) 0–300 V for 15 min; (2) 300 V for 45 min; 300 V to 3500 V for 3 h; 3500 V for 4 h. Focused strips were equilibrated in two steps of 15 min each with equilibration buffer [50 mM Tris–HCl, pH 8.8; 6M urea; 30% (*v*/*v*) glycerol and 2% (*w*/*v*) sodium dodecyl sulphate (SDS)], with the addition of 1% (*w*/*v*) DTT and 65mM iodoacetamide in the first and second steps, respectively. After equilibration the strips were applied in the top of a sodium dodecyl sulphate-polyacrylamide gel electrophoresis (SDS-PAGE) gel 14% acrylamide and run at 150 V constant voltage in a mini-protean system (BioRad, Hercules, CA, USA). Gels were stained with CBB-G250, as referred for SDS-PAGE gels. Gel images were acquired using a gel scanner (Epson) and Labscan software. ImageMaster 2D Platinum v7 software was used to analyse gel images. Spot editing and the match were performed automatically and corrected manually. Spot volume was normalized to the total spot volume. 

Spot identification was not performed specifically for the gels run in the present study, since equivalent spots have been previously identified by accurate mass-spectrometry based approaches in previous studies [20,21,22]. 

### 2.7. Statistical Analysis

Descriptive statistics was performed and data normal distribution and homoscedasticity were tested through Shapiro–Wilk and Levene tests, respectively. Salivary parameters were compared, within each treatment, between the periods before and after (or during, in the case of smell) stimulation through a *t*-test, when normality and homoscedasticity was achieved and through non-parametric approach (Mann–Whitney) when not.

To evaluate the existence of differences between periods, between stimuli and interaction period*stimuli, a two-way analysis of variance (ANOVA) repeated measures analysis within subjects was performed. The stimuli “bread odour, bread chewing and rice chewing” and periods “before and after” were considered as factors. 

All these statistical procedures were performed for saliva flow rate, total protein concentration, salivary amylase enzymatic activity and normalized spot volume. Statistical analysis was performed using SPSS v. 24, with significance level set at 5%.

## 3. Results

### 3.1. Salivary Flow Rate, Total Protein Concentration and Amylase Enzymatic Activity

Salivary flow rate was increased by all types of stimuli tested. Total protein concentration of saliva samples decreased when salivation was induced by odour, but did not change significantly due to bread or rice chewing. Concerning the enzymatic activity of salivary amylase, no changes were induced by any of the stimuli. Detailed results are presented in Table 1.

### 3.2. Salivary SDS-PAGE Protein Profile

**Figure 2:**
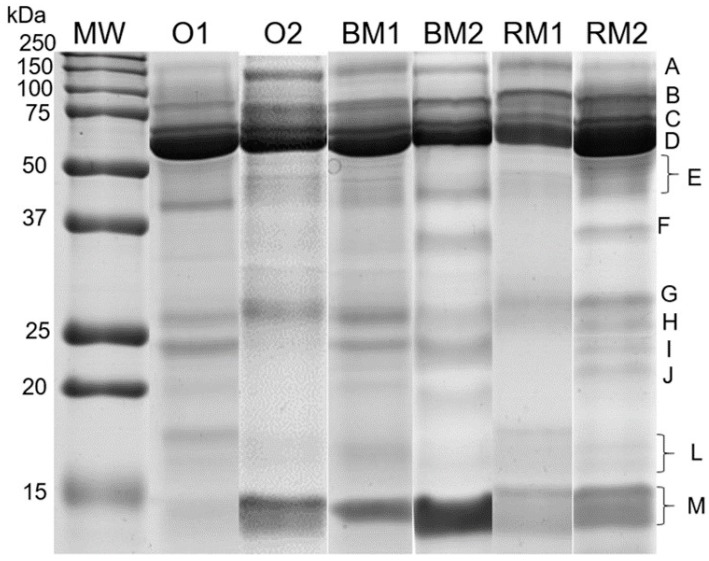
Sodium dodecyl sulphate-polyacrylamide gel electrophoresis (SDS-PAGE) salivary profiles representative of stimuli (O—bread odour; BM—bread mastication; RM—rice mastication); numbers represent saliva collection before (1) and after (2) stimuli.

Salivary SDS-PAGE protein profiles allowed the visualization of clearly distinct 12 protein bands (Figure 2). From these, 9 were present in all samples allowing comparison among stimuli (*N* = 25).

When analyzing the salivary protein profiles resultant from protein separation by SDS-PAGE, no major differences were observed between the effects produced by chewing the two different foods (bread and rice). Both with bread and rice mastication/ingestion, bands A, B, E and H decreased in their expression levels (Table 2). In the case of protein band A, which contains Ig polymeric receptor, this significant decrease in expression levels, induced by both types of mastication, was opposite to the effect of bread odour, which resulted in significant increases in the expression levels of this protein band. For the protein band H, also containing chains of immunoglobulins, although bread odour did not induce significant changes an interaction was observed among stimuli, suggesting a different effect when bread was smelled comparatively to when it was chewed (Table 2).

Bread and rice mastication/ingestion induced increases in the expression levels of band M, which is a band containing cystatins type S + cystatins type B. This was not observed when individuals smell bread, but a significant interaction among stimuli was observed, suggesting a different effect of smell or mastication/ingestion (Table 2).

### 3.3. Salivary Two-Dimensional Electrophoretic (2-DE) Profile

We used 2-DE profiles since they allow higher separation of salivary proteins in similar molecular masses range that SDS PAGE gels (Supplementary material). A total of 121 protein spots were consistently present in the salivary profiles, allowing statistical comparisons among stimuli and between periods (before and after stimulation) (N = 7).

**Figure 3:**
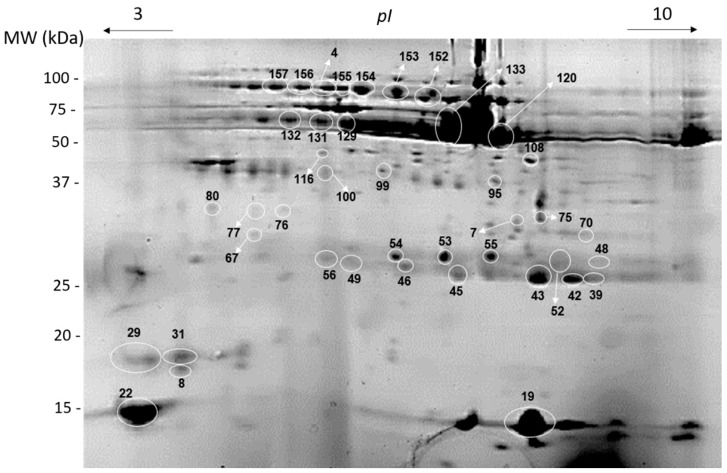
Representative 2-DE saliva protein profiles. Numbered spots are the ones differently expressed between stimuli and/or periods.

The different stimuli induced changes in salivary protein profile. Forty-one spots presented differences induced by the different stimuli (Figure 3; Table 3 and Table 4).

Bread odour-induced changes in the expression levels of 19 protein spots, from which 13 increased and 6 decreased (Table 3). Increases were observed for 2 spots of S-type cystatins, 7 spots of immunoglobulin chains, 2 spots of amylase, and 2 spots from non-identified proteins. By opposition, smelling bread reduced the expression levels of 2 spots of carbonic anhydrase VI (CA VI), 2 spots of Ig chains and 2 spots of proteins not identified. 

Concerning the effects of mastication/ingestion of bread or rice, some changes were common to both food products, whereas other changes were specific for each of them. Bread mastication resulted in the increase in levels of 2 spots of cystatins (spots 19 and 22, also increased with bread odour), 1 spot of prolactin-inducible protein, 1 spot of CA VI, 1 spot of short palate, lung, and nasal epithelial clone (SPLUNC), 2 spots of amylase (spots 120 and 133, also increased with bread odour) and 3 not identified spots. At the same time, bread mastication/ingestion resulted in the decrease of the expression levels of 15 protein spots, being all these spots already identified as chains of immunoglobulins.

Concerning rice mastication/ingestion, increases were observed for the expression levels of 2 spots of prolactin-inducible protein and 1 non-identified spot. In line with the changes induced by bread mastication, rice mastication also resulted in decreases in the expression levels of spots identified as chains of immunoglobulins (13 protein spots), most of which were the same observed to decrease with bread mastication/ingestion. Besides these, rice mastication/ingestion resulted in decreases in 1 SPLUNC spot and 2 not-identified spot. These results are detailed in Table 3 and Table 4.

## 4. Discussion

To the best of our knowledge, this is the first study which assessed the effect of food odour in salivary proteome. For this purpose, a combination of SDS-PAGE, which allowed protein separation in the total of participants, was combined with 2-DE for a deeper separation of the proteins, in a sub-sample. Recently, it was shown that salivary flow rate increases in response to food odours [9,10] and that increase was also observed in the present study, where smelling bread resulted in rise in the volume of saliva produced. This increase in salivation induced by food smelling is part of the cephalic phase response, with the function of preparing the body to optimize nutrient processing throughout the gastrointestinal tract [23].

When looking for specific effects of bread smelling at salivary protein level, a decrease in the total amount of protein secreted (total protein concentration) was observed, by contrast to what happened when solid food (bread or rice) were chewed. This decrease in total protein concentration can be due to a diluting effect of the augmented saliva volume. In the case of chewing, such a decrease was not observed. A recent review reported different findings among studies, concerning the effect of chewing on total protein concentration [24]. Some studies where non-taste materials, like parafilm, were chewed, reported decreases in total protein concentration [e.g., [25,26]], whereas a study where bread was chewed reported increases [27]. This indicates that the sum of mechanical stimulation, from chewing together with gustatory stimulation results in higher protein secretion. Masticatory-gustatory reflex of salivation consists in parasympathetic and sympathetic stimulation of salivary glands in response to gustatory stimulation on the one hand, and to somatosensory impulses on the other, resulting in increased secretion of both volume and protein amount [28]. This may explain why protein concentration is kept at similar levels than before chewing. 

Salivary alpha amylase is the protein usually associated with higher sympathetic stimulation of the parotid gland. The enzymatic activity of this protein was not affected by bread smelling, neither by bread or rice chewing/ingestion. The lack of increased salivary amylase enzymatic activity due to olfactory stimulation goes in line with results obtained by Morquecho-Campos et al. (2019), for different food stimuli. Amylase hydrolyses starch is responsible for the initial oral digestion of this food component. Since bread is a starch-rich product, a change in the levels of this protein in response to bread mastication/chewing could be expected. As such, the probability of a specific effect of starch level in inducing amylase secretion appears to be low. Amylase levels have been linked to the usual intake of starch [29,30], but this may imply a long-term regulation, different from that due to starch sensory signals. Studies about the effect of mastication in saliva secretion are not consensual for alpha-amylase secretion results: most authors found no changes after chewing [27,31,32], whereas some observed decreases [25,33], and others increases in its levels [34]. Further studies are needed to elucidate the mechanisms responsible for different amylase secretion in response to different sensory stimuli. 

The effect of olfactory stimulation in saliva secretion has been little studied. In these studies, it was concluded that only submandibular/sublingual glands respond to olfactory stimulation, with parotid having no response to olfactory stimuli [35,36,37]. Our work does not prove this concept but also does not deny it. Salivary proteins characteristic of submandibular/sublingual secretion, such as cystatins [38], increased with bread odor, but also with bread mastication/ingestion. This apparent increased stimulation of submandibular/sublingual glands can be due to olfactory stimuli, that are sensed both when individuals smell the bread and also when they chew the bread. In this last case, olfactory and gustatory cues will interact for the final sensory perception. Although in SDS-PAGE profiles the protein band identified as containing cystatins (band M) was also increased in response to rice mastication/ingestion, 2-DE profiles allow us to confirm that such increase was not due to the submandibular/sublingual specific S-type cystatins. This suggests that the stimulation of these glands by boiled rice was lower than the stimulation by bread. In fact, boiled rice does not have an intense smell, as bread does. 

Although salivary amylase enzymatic activity was not changed by bread smelling, as discussed above, and no significant variations were observed in the SDS-PAGE bands identified as containing salivary amylase, 2-DE profiles showed the increase in two amylase spots. Interestingly, these two spots also increased after chewing bread, but not after rice mastication/ingestion. Despite salivary amylase being secreted at higher levels by parotid glands, this protein is also secreted in submandibular/sublingual saliva, with no apparent differences in the forms that are secreted [38,39]. Although it is possible to hypothesize that these two spots increase in response to olfactory cues, which are also present when individuals chew the bread, it remains to be clarified why this happens only for these two amylase protein forms with no change in amylase enzymatic activity. Also, we cannot exclude that these common changes to smell and bread mastication/ingestion, that were observed for two amylase and two cystatin S spots, can be a specific response to bread. It was observed that cephalic phase responses in the secretion of insulin or the gastrointestinal peptide PP are nutrient specific, i.e., the response level is not equal for fat or carbohydrate stimuli, for example [40]. This may be due to the fact that in cephalic phase response the cortex is stimulated and sends messages for hypothalamus and/or amygdala and these latter send information to the parasympathetic nervous system through the vagus nerve [8]. This influence of higher brain areas in salivation has already been reported [41], reinforcing the possibility of different types of salivary secretions according to the type of stimulation. 

One of the most curious findings of this work was the opposite effect that food smelling and food mastication/ingestion seems to have in salivary immunoglobulin secretion. Both in SDS-PAGE and bi-dimensional electrophoretic profiles of saliva a decrease in the protein bands or spots of immunoglobulin chains with mastication/ingestion was evident. Bread odor, on the other hand, induced increased proportions of these proteins in saliva. The two principal antibody classes present in saliva are secretory IgA (sIgA) and IgG. IgM is also found in saliva. Whereas salivary IgA is derived from plasmatic cells, in the salivary glands, bound to secretory component in the membrane and secreted to saliva linked to it, IgG derives from blood circulation by passive leakage [42]. The reason why immunoglobulin chains are observed to decrease with bread or rice mastication must be probably the effect of chewing. Although Proctor and Carpenter reported increases in total sIgA with mastication [28], the effect in concentration (amount of protein per unit of volume) was reported to decrease [28]. Also, other authors observed decreased salivary immunoglobulin levels induced by the mechanical plus gustatory stimulation, characteristic of chewing [43,44]. 

The increase of immunoglobulins, caused by bread smelling, may be inconsistent if thinking that total volume secretion was significantly increased and that could cause dilution of salivary proteins. However, in line with what was stated above, this increase in immunoglobulin proportion, induced by bread smell, can result from the stimulation of submandibular/sublingual salivation by olfactory cues. In fact, it was reported that the submandibular glands contain, on average, approximately twice as many IgA+secretory components per tissue unit as the parotid [45]. Although further studies are necessary to understand the reason why an increase in these proteins levels occur in response to bread smelling, this effect of olfactory cues can be of clinical interest, helping the development of strategies to ameliorate oral immunological capacity. 

## 5. Conclusions

To our best knowledge, this is the first study showing the effect of food-olfactory stimulation in saliva proteome. Despite the recognized role that pre-ingestive signals may have in appetite/satiation and in the type and amount of food eaten, little attention has been paid to the effects of these signals in particular physiological aspects, such as salivation. Our results, besides confirming that smelling food results in augmented salivation, also show that saliva protein composition is also modified. Most of the changes that occurred when bread was smelled reinforce the previous suggestions of some authors arguing that olfactory food cues stimulate submandibular/sublingual glands, rather than the parotid. Even so, it would be important to explore in depth the effects of olfaction in saliva composition, since some of the changes were similar to those observed when the same food product was chewed/ingested, but not to those induced by the chewing/ingestion of a different type of food. This suggests that not all food odours have similar effects on saliva secretion, and these changes may be predicted by the type of food to be eaten, preparing saliva secretion and, ultimately, the gastrointestinal tract, to that particular food.

Another aspect particularly relevant was the opposite effect that olfactory cues had comparatively to food chewing/ingestion in what concerns salivary immunoglobulins. Bread smells induced increases in chains of immunoglobulins, suggesting that smelling food may have a positive effect on oral immunity. This may be particularly important from a clinical point of view, since salivary immunoglobulins are the first line components of an oral immunity system.

Finally, it is important to highlight that most of the salivary proteins affected by bread smelling are proteins involved in oral food perception. In the present work, an effect of these changes in in-mouth sensory perception of food was not tested. However, believing in the possibility that by changing the levels of the referred salivary proteins the sensory perception will also be changed, strategies for increasing the acceptance of some foods and decreasing others that are potentially unhealthy can become a possibility through smell interventions. This can be valuable for defining nutritional/dietary strategies or for the catering and food industry. Further studies, including those we are developing at the moment to access the effect of olfactory cues in subsequent food in-mouth sensory perception, are needed.

## Figures and Tables

**Table 1:** Variations in different salivary parameters induced by the odour or mastication (values are presented as mean ± standard deviation).

Salivary Parameter	Bread Odour		Bread Chewing		Rice Chewing	
	Before	After	Before	After	Before	After
Secretion rate (mL/min) (*N* = 25)	0.55 ± 0.33 ^a^	0.61 ± 0.37 ^b^	0.52 ± 0.27 ^a^	0.74 ± 0.40 ^b^	0.63 ± 0.31 ^a^	0.82 ± 0.44 ^b^
Protein concentration (µg/mL) (*N* = 25)	657.2 ± 412.9 ^a^	459.7 ± 201.5 ^b^	612.1 ± 307.0 ^a^	621.4 ± 240.4 ^a^	498.9 ± 225.8 ^a^	469.7 ± 236.1 ^a^
α-amylase (U/L) (*N* = 24)	126.6 ± 94.0	121.3 ± 97.8	135.1 ± 99.7	161.9 ± 87.1	133.9 ± 102.8	121.6 ± 106.0

Different upper letters mean significant differences between the periods before and after/during stimulation, within treatment.

**Table 2:** Expression levels (% volume; mean ± standard deviation) of the protein bands observed in SDS-PAGE salivary protein profiles, for each type of stimulation.

Protein Band	Protein	Assession Number (Uniprot)	MW (kDa) (Est/Theor.) #	Mascot ID Score	Sequence Coverage	Bread Odour		Bread Mastication		Rice Mastication		Interaction Period * Treatment *p*-Value
						Before	After	Before	After	Before	After	
A	Ig polymeric receptor + Lactotransferrin	P01833	125.0/84.4	107	21	5.32 ± 1.23 ^a^	6.04 ± 1.63 ^b^	6.02 ± 1.09 ^a^	4.76 ± 1.39 ^b^	5.55 ± 1.45 ^a^	4.39 ± 1.29 ^b^	<0.001 *
		P02788	125.0/80.0	89	20							
B	Serum albumin	P02768	71.0/71.3	109	19	8.86 ± 2.26	8.75 ± 2.48	8.91 ± 2.24 ^a^	7.78 ± 2.33 ^b^	8.12 ± 2.75 ^a^	7.00 ± 2.12 ^b^	0.457
C	α-Amylase 1	P04745	66.0/58.4	154	43	9.57 ± 2.75	9.81 ± 3.33	10.61 ± 3,69	8.85 ± 3,76	9.83 ± 3.23	8.68 ± 2.36	0.287
D	α-Amylase 1	P04745	60.0/58.4	100	21	14.90 ± 4.95	13.92 ± 4.69	14.53 ± 5.75	15.65 ± 5.61	15.65 ± 5.61	13.99 ± 4.91	0.913
E	Zinc-α2-glycoprotein + Carbonic anhydrase VI	P25311	41.0/34.5	71	30	9.99 ± 2.20	9.66 ± 1.93	9.68 ± 2.11 ^a^	8.48 ± 1.93 ^b^	9.79 ± 2.13 ^a^	8.49 ± 2.05 ^b^	0.308
		P23280	41.0/35.5	150	39							
H	Immunoglobulin kappa constant + Zymogen granule protein 16 homolog B	P01834	28.0/11.9	76	50	8.36 ± 2.15 ^a^	8.83 ± 2.25 ^a^	8.48 ± 2.55 ^a^	6.71 ± 2.05 ^b^	8.12 ± 2.56 ^a^	7.13 ± 2.57 ^a^	0.021 *
		Q96DA0	28.0/22.7	74	37							
I	Immunoglobulin kappa constant	P01834	23.5/11.9	74	50	7.41 ± 2.07	7.16 ± 2.20	7.19 ± 2.33	7.38 ± 1.95	7.56 ± 1.47	7.93 ± 1.30	0.616
L	Prolactin-inducible protein	P12273	16.5/16.8	131	60	5.85 ± 1.68	6.32 ± 2.01	6.18 ± 1.47	6.06 ± 1.60	6.01 ± 1.85	6.41 ± 2.18	0.785
M	Cystatin-SN	P01037	14.0/16.6	110	54	15.80 ± 4.55 ^a^	15.93 ± 4.14 ^a^	15.58 ± 3.87 ^a^	17.53 ± 4.20 ^b^	15.59 ± 3.12 ^a^	18.16 ± 5.41 ^b^	0.097
	Cystatin-S	P01036	14.0/16.5	109	58							

* Statistically significant for *p* < 0.05; Different upper cases in each column mean differences between periods before and after stimulation. # molecular masses (estimated/theoretical).

**Table 3:** Protein spots significantly changed by the different stimuli (values are mean ± standard error of % volume.

Spot	Bread Odour				Bread Mastication				Rice Mastication				^1^ Interaction Period * Treatment *p*-Value
	Before	After	*p*	Ratio (A/B) ^1^	Before	After	*p*	Ratio (A/B) ^1^	Before	After	*p*	Ratio (A/B) ^1^	
4	0.404 ± 0.171	0.503 ± 0.177	0.382	1.243	0.508 ± 0.164	0.377 ± 0.986	0.02 *	0.743	0.562 ± 0.136	0.357 ± 0.221	0.039 *	0.635	
7	0.063 ± 0.054	0.071 ± 0.053	0.768	1.127	0.117 ± 0.057	0.093 ± 0.038	0.482	0.794	0.092 ± 0.050	0.159 ± 0.056	0.049 *	1.735	
8	0.525 ± 0.349	0.551 ± 0.267	1.000	1.050	0.501 ± 0.152	0.680 ± 0.318	0.128	1.356	0.383 ± 0.171	0.633 ± 0.299	0.009 *	1.652	
19	2.692 ± 1.319	4.763 ± 2.249	0.009 *	1.769	2.326 ± 0.855	4.568 ± 2.357	0.03 *	1.964	3.130 ± 1.417	3.721 ± 1.161	0.306	1.189	
22	2.862 ± 1.637	5.456 ± 3.245	0.009 *	1.906	3.116 ± 1.681	5.319 ± 2.186	0.002 *	1.707	3.476 ± 1.573	3.968 ± 1.764	0.493	7.066	
29	0.384 ± 0.128	0.372 ± 0.087	0.808	0.968	0.410 ± 0.063	0.721 ± 0.133	0.004 *	1.757	0.549 ± 0.184	0.596 ± 0.233	0.634	1.087	0.03 *
31	0.476 ± 0.202	0.549 ± 0.290	0.518	1.154	0.655 ± 0.205	0.706 ± 0.220	0.675	1.078	0.452 ± 0.180	0.653 ± 0.172	0.046 *	1.443	
39	0.078 ± 0.048	0.772 ± 0.329	0.028 *	11.501	0.648 ± 0.163	0.309 ± 0.130	0.018 *	0.478	0.511 ± 0.343	0.364 ± 0.186	0.176	0.713	<0.001 *
42	0.101 ± 0.079	1.131 ± 0.900	0.022 *	11.188	0.970 ± 0.190	0.594 ± 0.194	0.003 *	0.613	0.817 ± 0.295	0.498 ± 0.104	0.054	0.610	0.002 *
43	0.641 ± 0.334	1.143 ± 0.491	0.117	1.785	1.330 ± 0.332	0.839 ± 0.282	0.003 *	0.631	1.200 ± 0.405	0.638 ± 0.233	0.013 *	0.531	0.004 *
45	0.561 ± 0.261	0.681 ± 0.338	0.444	1.214	0.783 ± 0.191	0.378 ± 0.163	0.0005 *	0.482	0.544 ± 0.150	0.506 ± 0.252	0.723	0.930	0.006 *
46	0.482 ± 0.311	0.338 ± 0.115	0.176	0.702	0.412 ± 0.220	0.225 ± 0.133	0.128	0.546	0.282 ± 0.066	0.125 ± 0.052	0.0005 *	0.443	
48	0.127 ± 0.082	0.245 ± 0.186	0.269	1.937	0.504 ± 0.171	0.253 ± 0.155	0.039 *	0.502	0.394 ± 0.218	0.119 ± 0.128	0.015*	0.301	0.001 *
49	0.317 ± 0.242	0.229 ± 0.177	0.542	0.723	0.494 ± 0.244	0.195 ± 0.209	0.009 *	0.394	0.188 ± 0.131	0.186 ± 0.146	0.981	0.990	
52	0.131 ± 0.110	0.411 ± 0.314	0.029 *	3.136	0.308 ± 0.147	0.274 ± 0.113	0.720	0.887	0.385 ± 0.287	0.098 ± 0.067	0.024 *	0.254	0.001 *
53	0.340 ± 0.189	0.656 ± 0.299	0.034 *	1.928	0.683 ± 0.237	0.431 ± 0.165	0.012 *	0.631	0.354 ± 0.090	0.400 ± 0.168	0.310	1.129	
54	0.513 ± 0.198	0.363 ± 0.240	0.045 *	0.708	0.348 ± 0.096	0.265 ± 0.071	0.119	0.762	0.297 ± 0.074	0.152 ± 0.060	0.016 *	0.512	
55	0.339 ± 0.119	0.548 ± 0.126	0.02 *	1.600	0.640 ± 0.124	0.424 ± 0.109	0.001*	0.663	0.436 ± 0.101	0.223 ± 0.144	0.002 *	0.512	0.006 *
56	0.546 ± 0.227	0.157 ± 0.151	0.011*	0.287	0.328 ± 0.161	0.168 ± 0.110	0.070	0.512	0.154 ± 0.183	0.074 ± 0.096	0.412	0.480	
67	0.083 ± 0.052	0.202 ± 0.208	0.179	2.434	0.100 ± 0.094	0.209 ± 0.093	0.019 *	2.102	0.169 ± 0.132	0.122 ± 0.096	0.238	0.723	
70	0.106 ± 0.078	0.220 ± 0.123	0.064	2.084	0.216 ± 0.169	0.170 ± 0.085	0.612	0.787	0.283 ± 0.215	0.122 ± 0.117	0.077	0.431	0.011 *
75	0.069 ± 0.055	0.142 ± 0.059	0.028 *	2.009	0.206 ± 0.132	0.204 ± 0.089	0.866	0.994	0.142 ± 0.043	0.077 ± 0.067	0.018 *	0.544	0.012 *
76	0.175 ± 0.062	0.113 ± 0.138	0.237	0.644	0.113 ± 0.036	0.080 ± 0.056	0.128	0.709	0.094 ± 0.035	0.058 ± 0.042	0.013 *	0.613	
77	0.091 ± 0.051	0.397 ± 0.312	0.075	4.050	0.131 ± 0.086	0.284 ± 0.085	0.018 *	2.175	0.129 ± 0.155	0.140 ± 0.147	0.753	1.195	
80	0.056 ± 0.055	0.404 ± 0.367	0.018 *	7.178	0.168 ± 0.123	0.419 ± 0.152	0.018 *	2.495	0.345 ± 0.213	0.268 ± 0.136	0.398	0.777	0.015 *
95	0.490 ± 0.262	0.089 ± 0.037	0.028 *	0.162	0.086 ± 0.104	0.221 ± 0.074	0.028 *	2.579	0.236 ± 0.160	0.083 ± 0.059	0.068	0.352	0.019 *
99	0.335 ± 0.243	0.065 ± 0.016	0.032 *	0.194	0.094 ± 0.060	0.156 ± 0.057	0.398	1.600	0.091 ± 0.020	0.114 ± 0.056	0.735	1.512	0.012 *
100	0.169 ± 0.160	0.140 ± 0.109	0.608	1.235	0.177 ± 0.084	0.257 ± 0.082	0.130	1.452	0.245 ± 0.083	0.143 ± 0.094	0.011 *	0.582	0.003 *
108	0.682 ± 0.293	0.166 ± 0.147	0.008 *	0.243	0.107 ± 0.073	0.209 ± 0.084	0.013 *	1.953	0.091 ± 0.043	0.098 ± 0.064	0.735	1.077	0.045 *
116	0.155 ± 0.082	0.043 ± 0.030	0.037*	0.277	0.028 ± 0.009	0.062 ± 0.017	0.004 *	2.198	0.044 ± 0.028	0.074 ± 0.040	0.114	1.684	0.019 *
120	0.096 ± 0.060	2.308 ± 1.602	0.019 *	24.042	1.723 ± 0.945	2.462 ± 0.811	0.018*	1.428	2.625 ± 1.676	1.793 ± 0.859	0.261	0.6830	
129	0.737 ± 0.707	1.732 ± 1.217	0.091	2.348	1.837 ± 0.373	0.979 ± 0.390	0.028 *	0.533	0.698 ± 0.297	0.622 ± 0.209	0.866	0.892	0.005 *
131	0.072 ± 0.058	0.871 ± 0.474	0.01 *	8.736	1.335 ± 0.680	0.893 ± 0.235	0.083	0.669	1.207 ± 0.400	0.688 ± 0.322	0.017 *	0.570	0.002 *
132	1.143 ± 0.547	1.141 ± 0.676	0.994	0.998	1.392 ± 0.499	0.692 ± 0.172	0.004 *	0.497	1.292 ± 0.879	0.888 ± 0.464	0.174	0.918	
133	0.820 ± 0.210	3.580 ± 0.977	0.028 *	4.123	4.767 ± 1.911	0.959 ± 0.460	0.043 *	1.510	3.819 ± 0.601	2.930 ± 1.162	0.063	0.794	0.01 *
152	0.043 ± 0.112	0.503 ± 0.244	0.003 *	11.698	0.779 ± 0.264	0.439 ± 0.169	0.024 *	0.564	0.510 ± 0.197	0.296 ± 0.099	0.032 *	0.580	0.002 *
153	0.433 ± 0.288	0.654 ± 0.256	0.264	1.980	0.713 ± 0.189	0.478 ± 0.175	0.012 *	0.671	0.623 ± 0.200	0.404 ± 0.236	0.062	0.649	0.011 *
154	0.417 ± 0.041	0.589 ± 0.240	0.612	1.307	0.924 ± 0.492	0.484 ± 0.115	0.028 *	0.524	0.693 ± 0.297	0.399 ± 0.229	0.018 *	0.576	
155	0.450 ± 0.115	0.400 ± 0.297	0.678	0.889	0.690 ± 0.322	0.446 ± 0.876	0.047 *	0.646	0.665 ± 0.158	0.430 ± 0.243	0.027 *	0.647	0.005 *
156	0.436 ± 0.132	0.411 ± 0.257	0.810	0.944	0.491 ± 0.251	0.320 ± 0.047	0.096	0.651	0.475 ± 0.140	0.304 ± 0.146	0.046 *	0.641	0.023 *
157	0.301 ± 0.093	0.345 ± 0.185	0.548	1.146	0.421 ± 0.196	0.266 ± 0.050	0.058	0.632	0.386 ± 0.119	0.273 ± 0.110	0.047 *	0.709	0.047 *

^1^ Statistically significant interaction between treatment and period; * significant at *p* < 0.05.

**Table 4:** Identification and location in the 2-DE profiles of the salivary proteins significantly changed by stimuli.

Spot	Protein Identification	Apparent Isoelectric Point (pI)	Apparent Molecular Mass (kDa)	Ref (Identifying the Proteins by Mass Spectrometry)
**4**	Ig polymeric receptor	5.7	100	[20,21,22]
**7**	n.i.	6.8	32	
**8**	Prolactin inducible protein	4.5	18	[21]
**19**	Cystatin SA	7.1	15	[22]
**22**	Cystatin S	4.0	15	[21]
**29**	Prolactin inducible protein	4.0	19	[20,22]
**31**		4.7	19	[20,21,22]
**39**	Ig Kappa chain reaction	8.3	27	[20,21,22]
**42**		7.7	27	
**43**		7.1	28	
**45**		7.0	28	
**46**		6.8	29	
**48**		8.3	29	
**49**		5.8	29	
**52**		7.8	29	
**53**		6.1	30	
**54**		5.9	30	
**55**		6.9	29	
**56**	n.i.	5.7	29	
**67**	n.i.	5.2	31	
**70**	n.i.	8.3	31	
**75**	n.i.	7.3	33	
**76**	SPLUNC	5.4	33	[20,22]
**77**		5.3	33	
**80**	n.i.	5.0	34	
**95**	carbonic anhydrase VI	6.9	42	[22]
**99**		5.9	45	
**100**	n.i.	5.7	44	
**108**	n.i.	7.3	47	
**116**	n.i.	5.7	49	
**120**	α-amylase	7.0	60	[19,21]
**129**	Ig alpha-1 chain C region	5.8	66	[15]
**131**		5.7	66	
**132**		5.6	68	
**133**	α-amylase	6.3	68	[19,21]
**152**	Ig polymeric receptor	6.3	89	[20,21,22]
**153**		6.2	93	
**154**		5.9	96	
**155**		5.8	100	
**156**		5.7	100	
**157**		5.6	100	

## Supplementary Materials

The following are available online at https://www.mdpi.com/2072-6643/12/4/1002/s1, Figure S1: Gel images of individual saliva samples collected before bread smelling; Figure S2: Gel images of individual saliva samples collected during bread smelling; Figure S3: Gel images of individual saliva samples collected before bread chewing; Figure S4: Gel images of individual saliva samples collected after bread smelling; Figure S5: Gel images of individual saliva samples collected before rice chewing; Figure S6: Gel images of individual saliva samples collected after rice chewing.
