# Supplementary figures and images for: Changes in Salivary Proteome in Response to Bread Odour
Source: Nutrients. 2020 Apr 5;12(4):1002. doi: 10.3390/nu12041002 (PMC7230670; doi:10.3390/nu12041002)

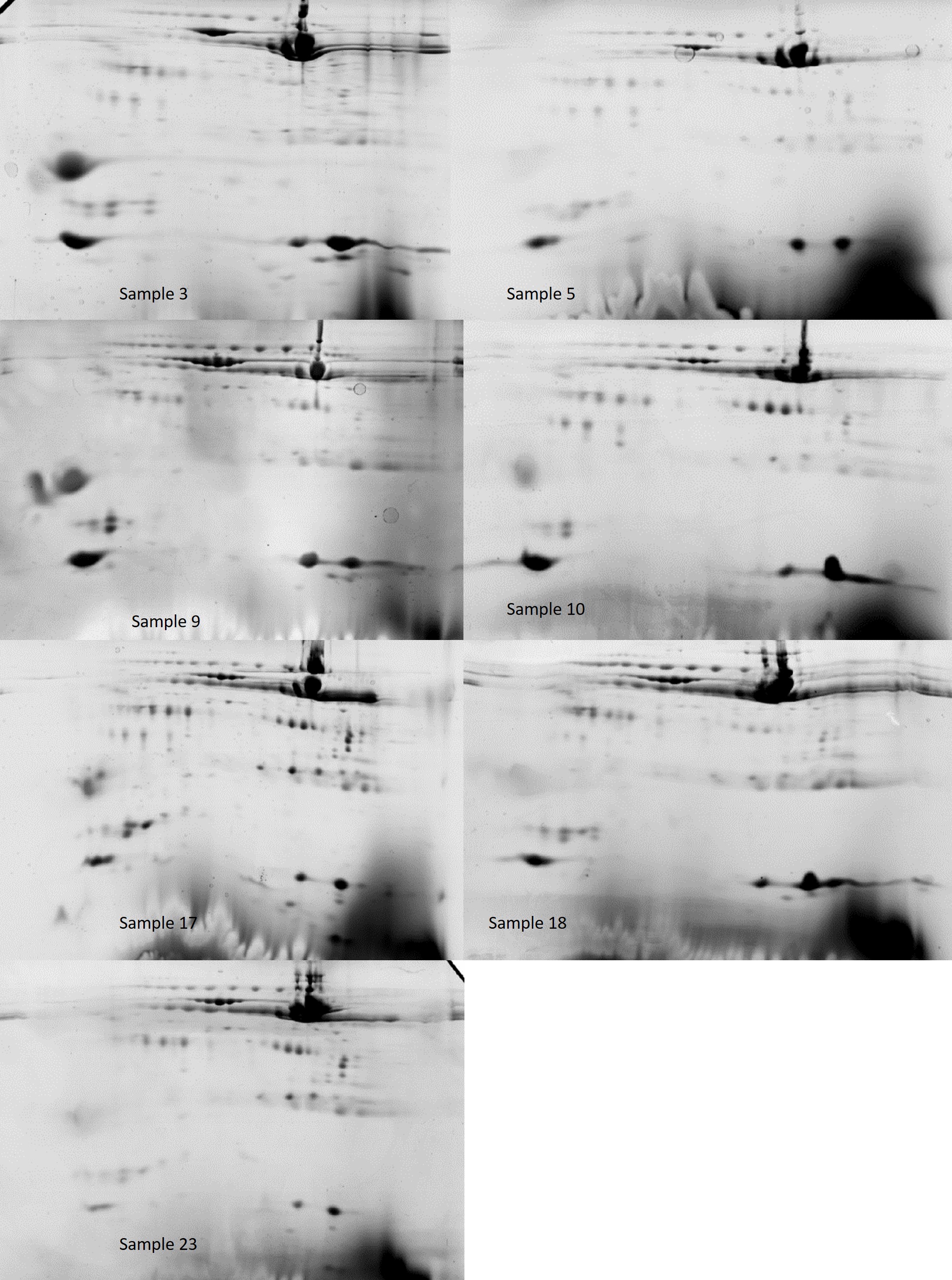

Supplement: Supplementary file 1 [file nutrients-12-01002-s001.zip › Supp_bread chewing after.jpg]

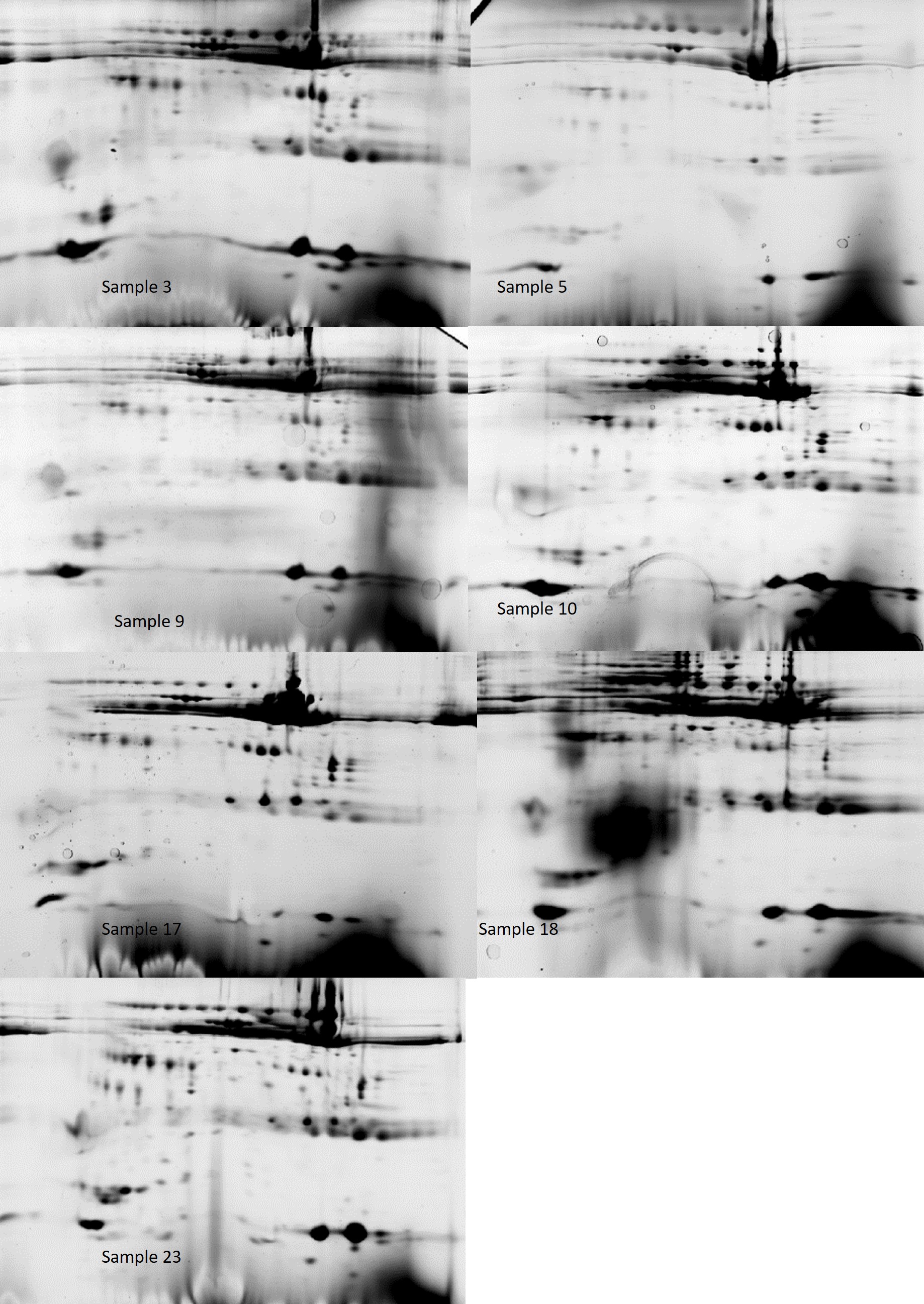

Supplement: Supplementary file 1 [file nutrients-12-01002-s001.zip › Supp_bread chewing before.jpg]

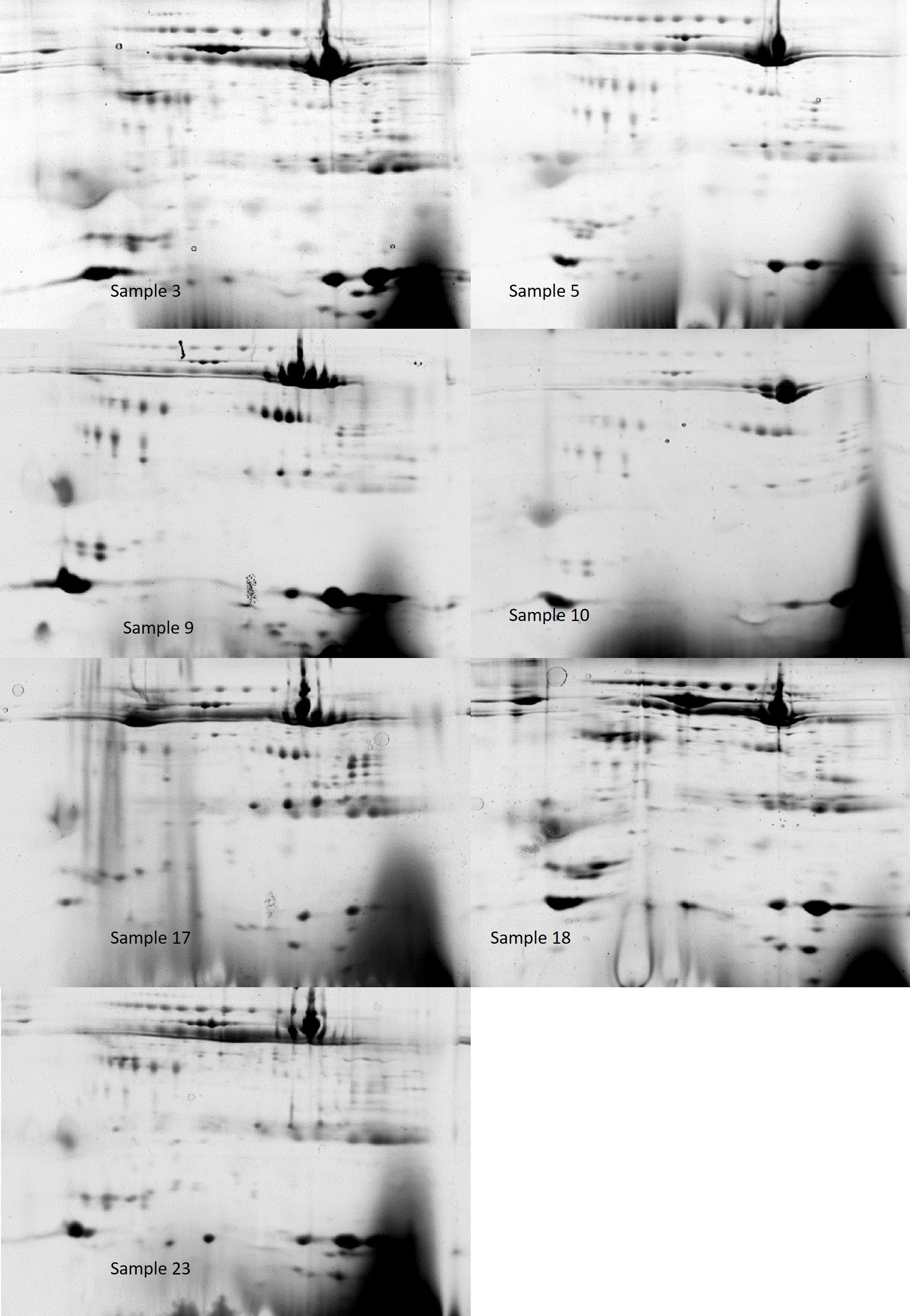

Supplement: Supplementary file 1 [file nutrients-12-01002-s001.zip › Supp_odor_after.jpg]

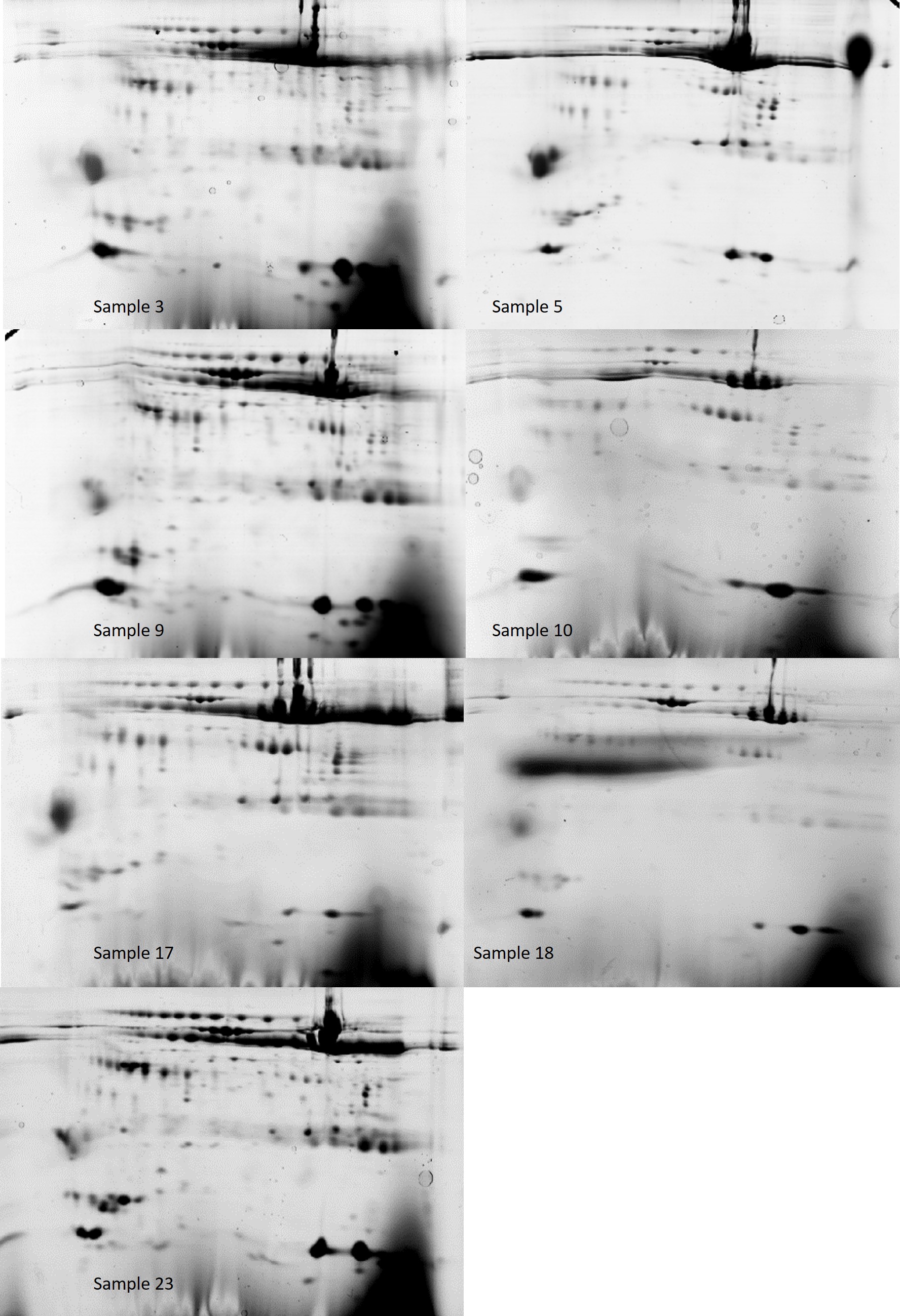

Supplement: Supplementary file 1 [file nutrients-12-01002-s001.zip › Supp_odor_before.jpg]

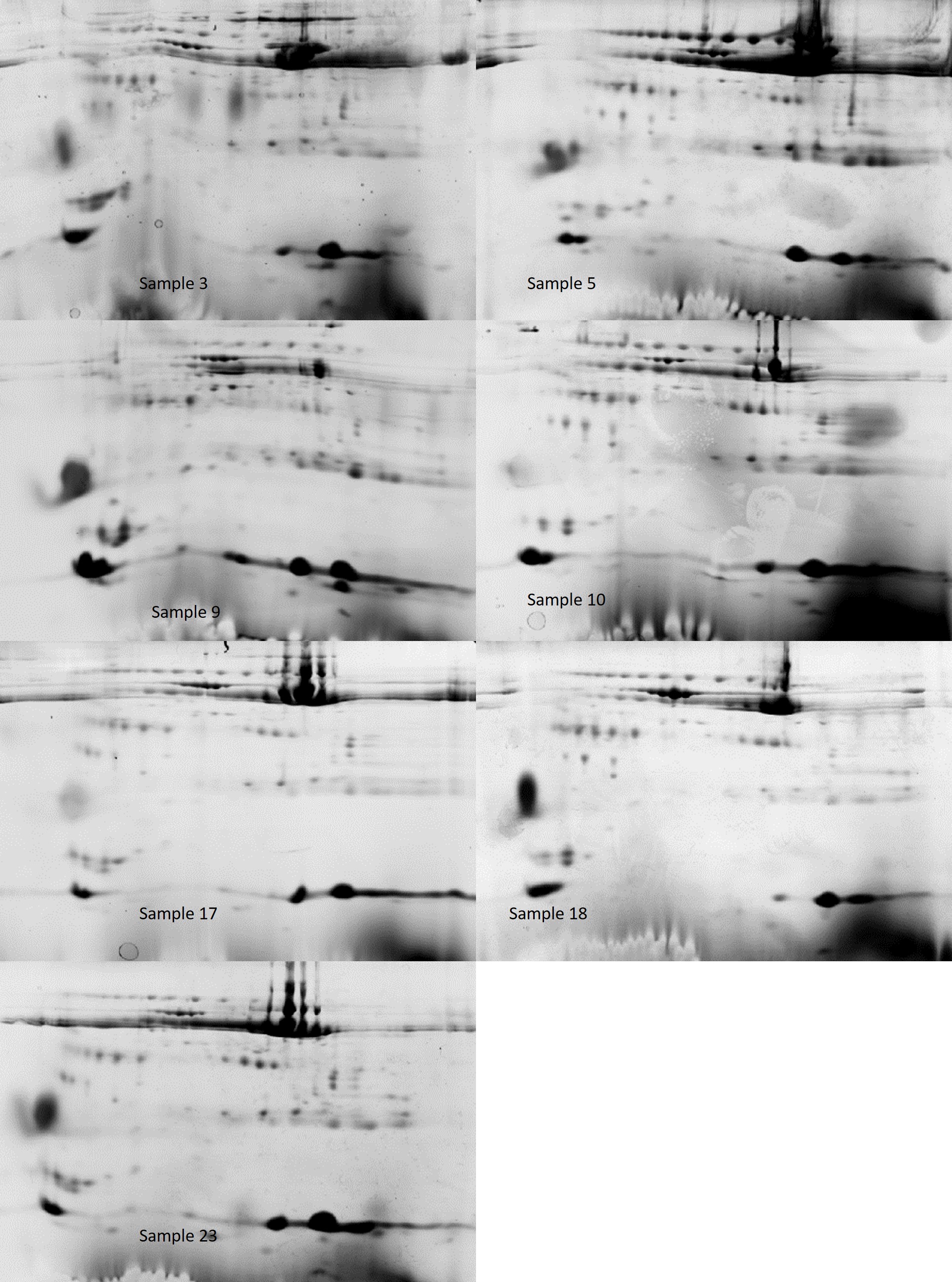

Supplement: Supplementary file 1 [file nutrients-12-01002-s001.zip › Supp_rice chewing after.jpg]

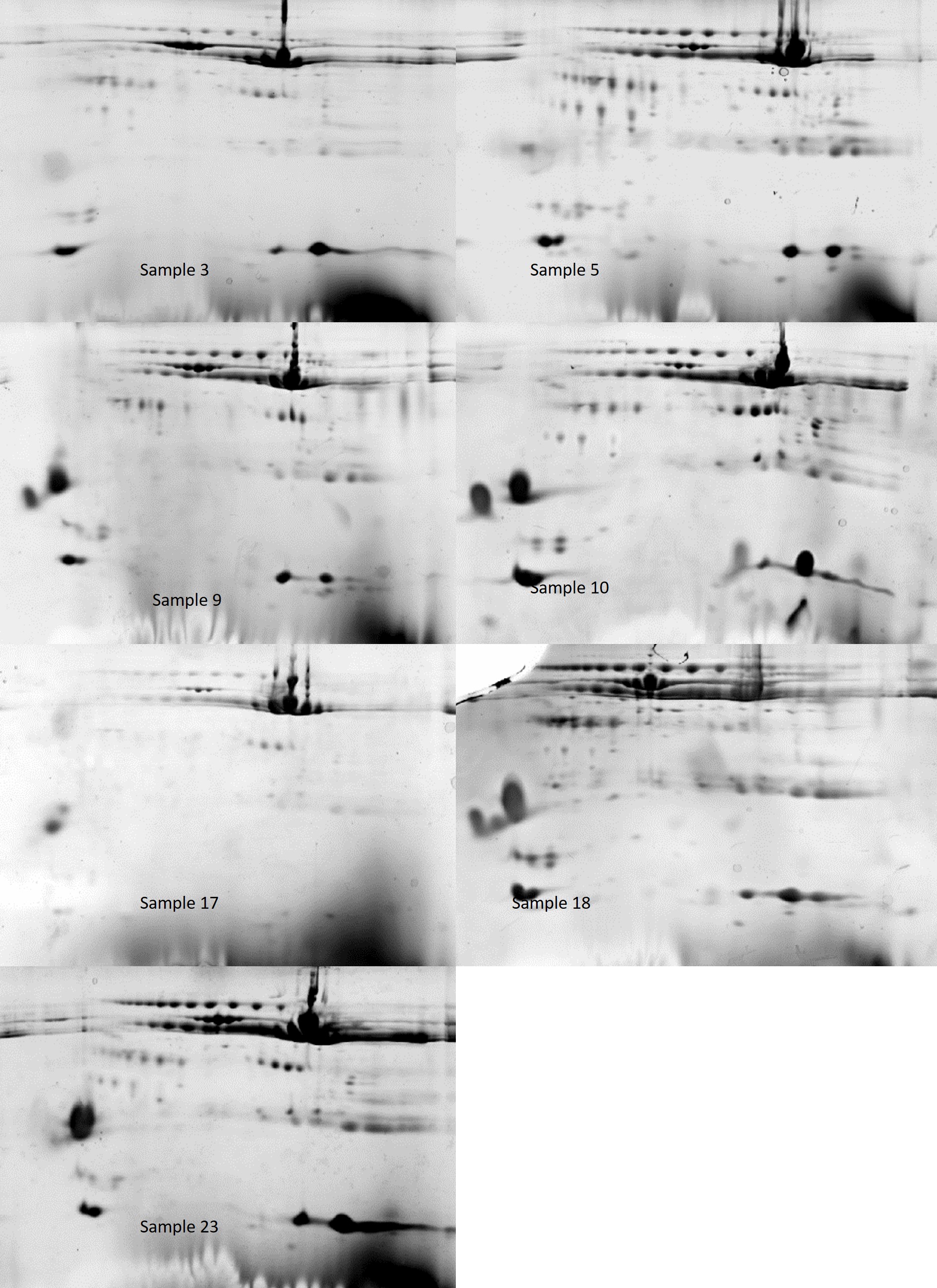

Supplement: Supplementary file 1 [file nutrients-12-01002-s001.zip › Supp_rice chewing before.jpg]
